# Quantitative assessment of choroidal parameters and retinal thickness in central serous chorioretinopathy using ultra-widefield swept-source optical coherence tomography: a cross-sectional study

**DOI:** 10.1186/s12886-024-03405-w

**Published:** 2024-04-17

**Authors:** Bei Xiao, Ming Yan, Yan-Ping Song, Ya Ye, Zhen Huang

**Affiliations:** 1 The First School of Clinical Medicine, Southern Medical University, 510515 Guangzhou, China; 2 The Department of Ophthalmology, General Hospital of Central Theater Command, No. 627 Wuluo Road, Wuchang District, 430000 Wuhan, Hubei Province China

**Keywords:** Central serous chorioretinopathy (CSC), Choroid, Swept-source optical coherence tomography angiography (SS-OCTA), Three-dimensional choroidal vessel index

## Abstract

**Objective:**

In this study, we used ultra-wide field swept-source optical coherence tomography angiography (UWF SS-OCTA) to assess changes in retinal thickness (RT) and choroidal parameters in individuals who had received a diagnosis of central serous chorioretinopathy (CSC).

**Methods:**

The study encompassed the evaluation of 59 eyes from 47 patients with a diagnosis of CSC, alongside 33 fellow eyes and 31 eyes from healthy individuals (controls). The parameters assessed included RT, choroidal thickness (CT), choriocapillaris density, vascular density of the large choroidal vessel layer, three-dimensional choroidal vascularity index (3D-CVI), choroidal vessel volume per unit area (mCVV/a), and choroidal stroma volume per unit area (mCSV/a).

**Results:**

Metrics including mCVV/a, mCSV/a, 3D-CVI, CT, and RT exhibited significantly elevated values in the eyes affected by CSC compared to those of the control group across nine subfields. Moreover, a substantial number of the subfields in both CSC-affected and fellow eyes exhibited increased values for mCVV/a, mCSV/a, 3D-CVI, CT, and RT when compared with the control group. Additionally, acute and chronic CSC subfields demonstrated significantly elevated values for mCVV/a, mCSV/a, 3D-CVI, CT, and RT in comparison to healthy control eyes. Notably, specific subfields associated with complex and atypical forms of CSC revealed higher metrics compared to those of the control group.

**Conclusion:**

In conclusion, the UWF SS-OCTA proved to be a valuable tool for exploring the anatomical etiology and clinical classification and diagnosis of CSC.

**Supplementary Information:**

The online version contains supplementary material available at 10.1186/s12886-024-03405-w.

## Introduction

Central serous chorioretinopathy (CSC) constitutes a pathological condition that impacts the choroidal retina, characterized by a dysfunction in the retinal pigment epithelium (RPE) [1]. The conventional classification of CSC into acute (≤ 6 months) and chronic (> 6 months) forms, although commonly used [2], often proves to be fraught with ambiguity in practical application owing to subjective symptomatology reported by patients and inconsistencies in case selection methodologies across various studies [3–6]. In 2020, the CSC International Group introduced a novel classification system for CSC grounded in multimodal imaging techniques [7]. Nevertheless, there is a scarcity of research exploring the choroidal aspects of CSC using this multimodal imaging-based classification system.

Diagnostic imaging plays an integral role in the assessment of CSC. Optical coherence tomography angiography (OCTA) represents an innovative imaging modality that capitalizes on blood flow signals, providing a detailed visualization of ocular vasculature without the need for contrast media. Among various OCTA variants, swept-source OCTA (SS-OCTA) stands out due to its enhanced imaging speed, deeper penetration capabilities, and reduced susceptibility to interference from refractive media. Ultra-wide field SS-OCTA (UWF SS-OCTA) has emerged as an indispensable tool for assessing peripheral choroidal changes in eyes impacted by CSC [8–10]. Previous studies have utilized OCT to scrutinize changes in the RPE and the outer retina in cases of CSC. These studies have been instrumental in assessing the presence of subretinal fluid (SRF), a critical parameter for determining the duration of the disease and formulating appropriate therapeutic strategies [8–11]. While the role of the choroid in CSC pathogenesis, particularly choroidal thickening and hyperpermeability, is well-established [12, 13], prior research efforts have often neglected to examine choroidal changes beyond the macular region.

In the present study, we delve deeper into choroidal modifications, including choroidal metrics and retinal thickness (RT) in patients with CSC by employing UWF SS-OCTA with a scanning scope of 24 mm × 20 mm. To the best of our knowledge, no previous study has harnessed blood flow and thickness indices from UWF SS-OCTA to distinguish between CSC subtypes. Such a discrimination is of pivotal significance for guiding therapeutic decision-making and estimating prognosis. Consequently, we seek to advance the diagnosis and classification of CSC from an imaging perspective by leveraging UWF SS-OCTA indices, thereby enhancing the understanding of the anatomical etiology of CSC and expanding the applications of OCTA in the context of CSC pathology.

## Materials and methods

### Study participants

This prospective observational study was conducted by the Department of Ophthalmology of the General Hospital of Central Theater Command from April to August 2023. We enrolled 47 patients (59 eyes) who had been diagnosed with CSC. Additionally, 33 fellow eyes without CSC and 16 sex- and age-matched individuals with healthy eyes (31 eyes) were incorporated as controls. Eligibility for participation required a confirmed ocular diagnosis of central serous chorioretinopathy for the CSC-affected individuals. This study was approved by the institutional ethics review committee of the General Hospital of Central Theater Command and conducted in accordance with the principles outlined in the Declaration of Helsinki. Before receiving any sort of treatment, all participants provided a signed informed consent form. All the participants underwent a full examination including a detailed history assessment. This encompassed the duration of symptoms, age, gender, ocular gender, best-corrected visual acuity (BCVA), and prior medical history of hypertension and diabetes mellitus.

### Inclusion and exclusion criteria

CSC diagnosis relied on the following criteria: (1) Presence of SRF and/or pigment epithelial detachment (PED) as detected by SS-OCT; (2) Apparent dye leakage from the RPE on fundus fluorescein angiography (FFA); (3) Enhanced focal choroidal vascular hyperpermeability detected on indocyanine green angiography(ICGA) during the late phase.

Participants were excluded based on the following criteria: (1) The presence of myopia characterized by significant media opacities and refractive errors exceeding 6 diopters in spherical magnitude or 3 diopters in cylindrical magnitude, which could potentially impede the acquisition of clear images; (2) Factors that compromise the quality of imaging (OCT or OCTA quality score below 8), including conditions such as cataracts, inadequate fixation, or substantial refractive media opacities that prevent the participant from undergoing the examination; (3) Prior ocular procedures performed internally, excluding cataract surgery; (4) Severe systemic diseases.

Participants underwent further classification into either acute or chronic CSC based on a retrospective analysis of historical data and the observations derived from imaging assessments conducted during their outpatient visits. In line with the new multimodal imaging-based classification system introduced by the CSC International Group in 2020, each case was classified as follows: (a) Simple CSC: Spontaneous fluorescence (FAF) abnormalities on the retinal pigment epithelium (RPE) ≤ 2 disc areas (DA); (b) Complex CSC: FAF abnormalities on RPE > 2 DA; (c) Atypical CSC: Bullous variant, RPE tear, association with other retinal diseases. The classification standards of simple, complex, and atypical CSC are obtained from the study by Chhablani et al. [14].

### Ultra-widefield swept source optical coherence tomography analysis

**Fig. 1:**
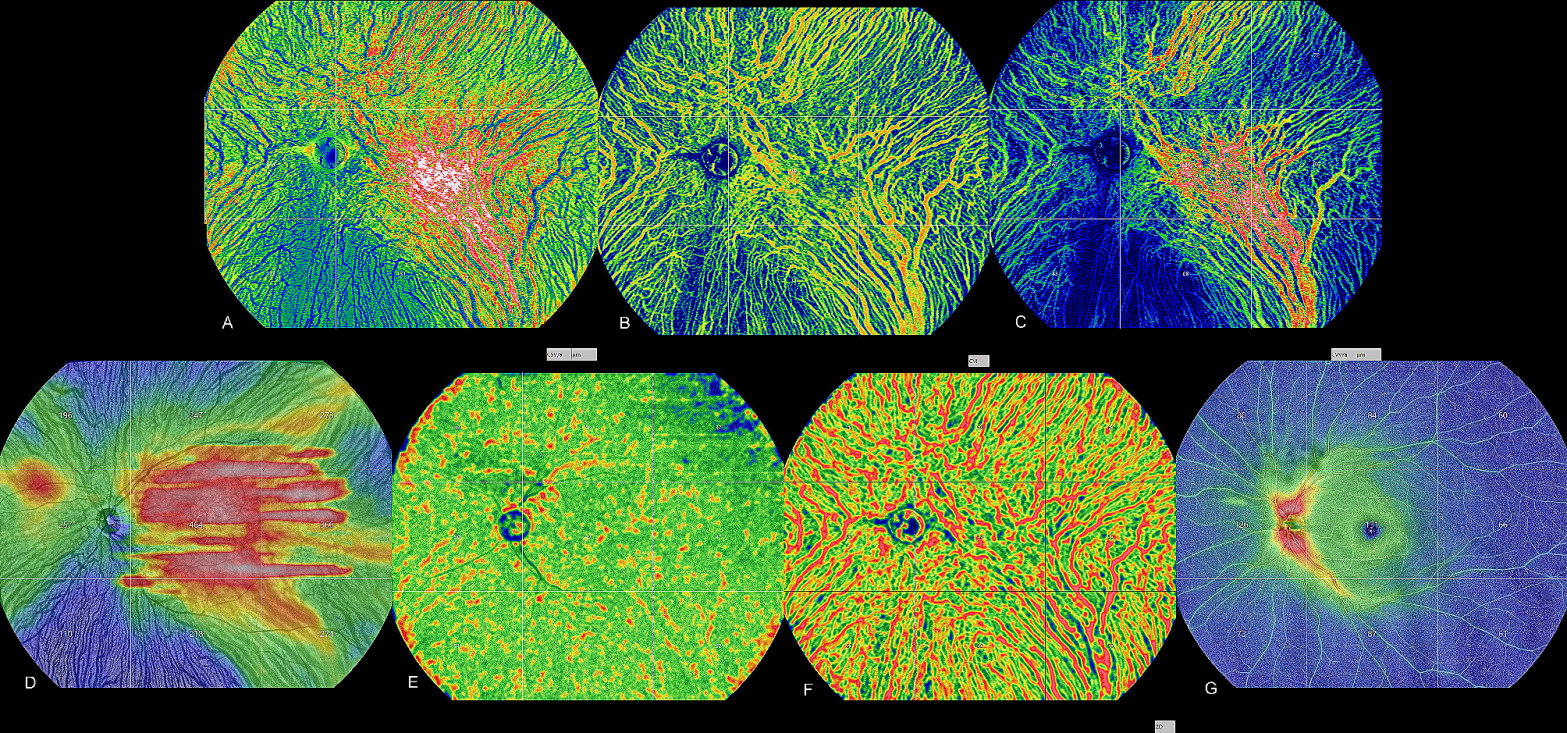
Ultra-widefield swept-source optical coherence tomography (UWF SS-OCTA) images that are representative (**A**) Choroid vessel volume per unit area (mCVV/a) across nine subfields (**B**) Three-dimensional choroidal vessel volume index (3D-CVI) across nine subfields (**c**) Choroid stroma volume per unit area (mCSV/a) across nine subfields (**D**) Choroidal thickness (CT) across nine subfields (E) Vascular density of the choriocapillaris layer across nine subfields (**F**) Vascular density of the large choroidal vesasel layer across nine subfields (**G**) Retinal thickness (RT)

The participants underwent imaging using an SS-OCTA device with a 400 kHz A-scan rate, model BM-400 K BMizar (TowardPi Medical Technology, Beijing, China). The specific instrument settings were 400 kHz A-scan rate, axial scan depth of 6 mm, B-scan length of 24 mm, and OCTA imaging area measuring 24 × 20 mm, centered on the fovea. Additionally, structural OCT was employed to visualize cross-sectional macular structures. The accompanying software delineated nine subfields, which included superotemporal, upper, superonasal, temporal, central, nasal, inferotemporal, lower, and inferonasal regions, for the purpose of recording various parameters, including choroidal thickness (CT), choriocapillaris density, vascular density of the large choroidal vessel layer, three-dimensional choroidal vascularity index (3D-CVI), choroidal vessel volume per unit area (mCVV/a), choroidal stroma volume per unit area (mCSV/a), and RT. The vascular density of the large choroidal vessel layer was determined by calculating a density value based on traditional 2D enface images. Choriocapillaris density and the vascular density of the large choroidal vessel layer were automatically computed as the ratio of vessel pixel areas to the total region area. The large-vessel choroidal layer was defined as the slab located between the choroid-scleral interface (CSI) and 29 mm beneath Bruch’s membrane (BM). The choroidal vessel volume per unit area index (CVI) represented the ratio of choroidal vessel volume to choroid volume within a specified three-dimensional region, which is equivalent to a 3D vessel density. The metrics mCVV/a and mCSV/a quantified choroidal vessel volume and choroidal stroma volume per unit area, respectively. RT was defined as the distance between the inner limiting membrane (ILM) and the outer plexiform layer (OPL), while CT was determined as the distance from BM to the CSI. Both BM and CSI were automatically identified by the software, and, if necessary, their accuracy was manually verified using B-scans. To ensure standardized measurements among participants, adjustments were made to account for the AL-related magnification using the modified Littmann formula (Bennett procedure). The measurement position consistently centered on the fovea without rotation, and data from the left eyes were horizontally inverted for statistical analysis (Fig. 1).

### Statistical analysis

Statistical analysis was performed using IBM SPSS software version 27.0. Data sets conforming to a Gaussian or normal distribution are presented as mean ± standard deviation (SD). The normality of the data distribution was assessed employing the Kolmogorov–Smirnov test. In the case of participant attributes, we applied descriptive techniques, and data were expressed using conventional summary statistics, including mean (SD), median, interquartile range (IQR), and proportional values.

To determine the relationship between the CSC-affected eye and its fellow, the healthy eye and the CSC-affected eye, and the fellow eye and the healthy eye, data that followed a normal distribution were assessed using ANOVA, while non-normally distributed data were assessed using the Kruskal-Wallis signed rank test. For comparisons between acute CSC and chronic CSC, chronic CSC and healthy eyes, and acute and chronic CSC, data that followed a normal distribution were assessed using ANOVA, whereas those deviated from a normal distribution were assessed using the Kruskal–Wallis signed rank test. For comparisons between eyes with simple CSC, complex CSC, atypical CSC, and healthy eyes, data that followed a normal distribution were examined using the independent *t*-test, while those that did not follow a normal distribution were analyzed using the Wilcoxon rank-sum test.

We employed receiver operating characteristic (ROC) curve analysis to determine the orientation of the most effective diagnostic signs for acute and chronic CSC-affected eyes. A *p*-value less than 0.05 was deemed statistically significant.

## Results

### Baseline data

The study cohort comprised 47 patients diagnosed with CSC, consisting of 35 males and 12 females. Additionally, 33 fellow eyes were included in the study, comprising of 23 males and 10 females, while 31 healthy participants (controls) were part of the analysis, comprising of 28 males and 3 females. A comprehensive and detailed summary of participant characteristics can be found in Table 1.

**Table 1:** Characteristics of patients with CSC

Clinical parameters	Patients with CSC(*n* = 47)	No.(%)
Age(years)		
< 50	29.00	61.70
≥ 50	18.00	38.30
Gender		
male	35.00	74.47
female	12.00	25.53
suffering from eye disease		
single	34.00	72.34
both	13.00	27.66
first episode		
Yes	13.00	27.66
No	34.00	72.34
Diabetes		
Yes	6.00	12.77
No	41.00	87.23
Hypertension		
Yes	8.00	17.02
No	39.00	82.88

### Comparison of choroidal parameter and retinal thickness in nine subfields in the CSC-affected eyes, the fellow eye, and healthy eyes

A total of 24 cases were diagnosed with acute CSC, while chronic CSC was identified in 35 cases. Among these, 34 cases were categorized as simple CSC, 15 as complex CSC, and 10 as atypical CSC.

**Fig. 2:**
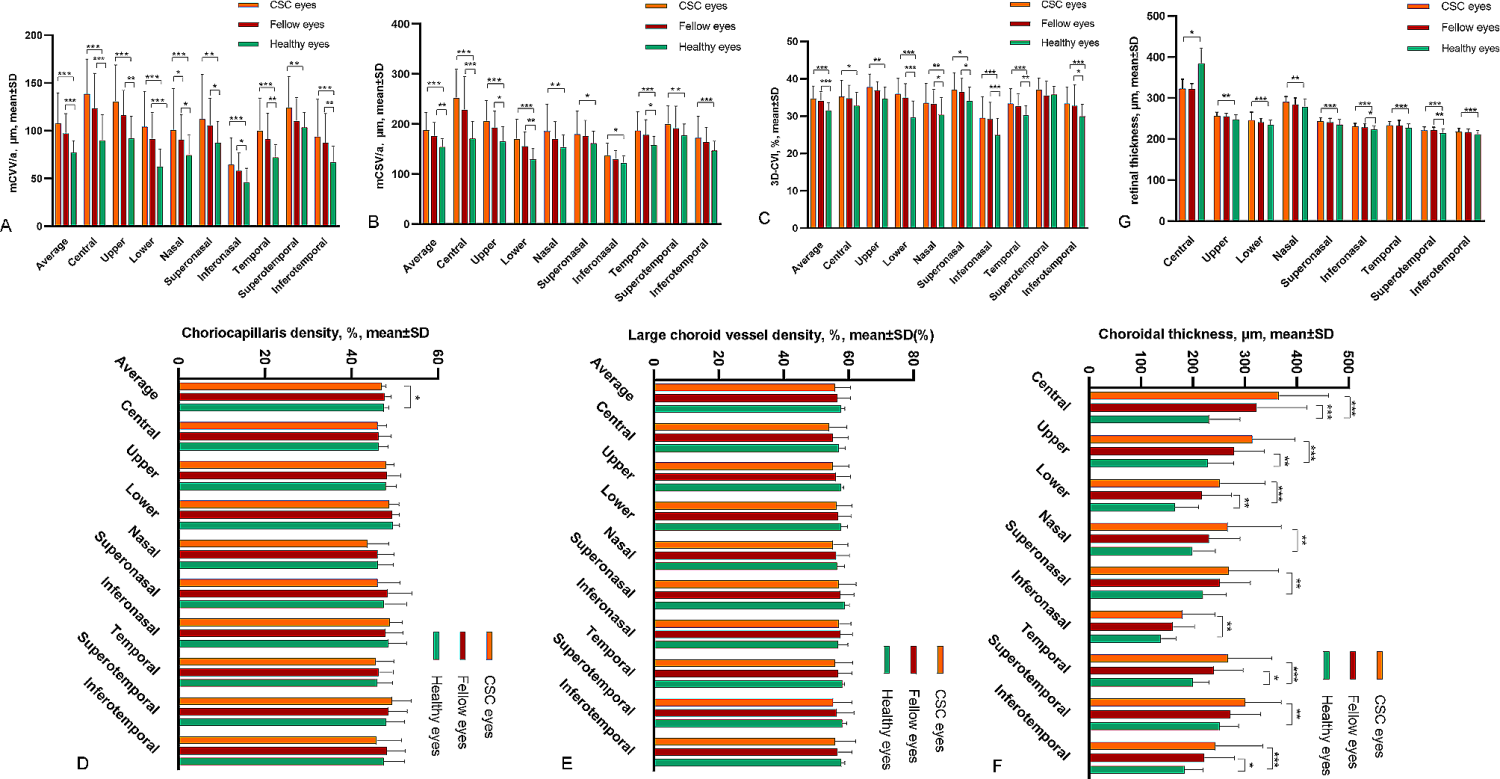
Choroidal characteristics and retinal thickness were assessed in nine subfields among eyes with CSC, fellow eyes, and healthy eyes (**A**) Choroidal vessel volume per unit area (mCVV/a); (**B**) Choroidal stroma volume per unit area (mCSV/a); (**C**) Three-dimensional choroidal vascularity index (3D-CVI); (**D**) Choriocapillaris density; (**E**) Large choroid vessel density; (**F**) Choroidal thickness; (**G**) Retinal thickness. ∗*P* < 0.05; ∗∗*P* < 0.01; ∗∗∗*P* < 0.001

Figure 2 presents an overview of the data for nine distinct subfields, encompassing various parameters such as CT, choriocapillaris density, vascular density of the large choroidal vessel layer, 3D-CVI, mCVV/a, mCSV/a, and RT, which were assessed for the eyes affected by CSC, fellow eyes, and healthy eyes (for detailed information, refer to Supplementary Table 1).

Notably, mCVV/a in CSC-affected eyes exhibited significantly higher values in the nasal regions (100.85 ± 43.2 μm) compared to fellow eyes (90.41 ± 26.73 μm, *P* = 0.041). This difference was statistically significant. Furthermore, when compared to healthy eyes, mCVV/a in CSC-affected eyes displayed significantly elevated values in all nine subfields, indicating a significant difference (*P* < 0.01). In contrast, mCVV/a in fellow eyes revealed significant differences in most regions compared to healthy eyes, with the exception of the superotemporal areas.

In contrast, mCSV/a in CSC-affected eyes did not exhibit any significant differences across all subfields when compared to fellow eyes (*P* > 0.05). However, when compared with healthy eyes, mCSV/a in CSC-affected eyes displayed significantly higher values across all subfields (*P* < 0.05). Conversely, mCSV/a in fellow eyes demonstrated a significant increase compared to healthy eyes in specific regions, namely the upper, temporal, central, lower, and on average.

No significant differences were observed in 3D-CVI across all subfields between CSC-affected eyes and fellow eyes (*P* > 0.05). In comparison to healthy eyes, significant differences in 3D-CVI were evident in CSC-affected eyes across most subfields, except for the superotemporal regions. Additionally, 3D-CVI in fellow eyes surpassed the values observed in healthy eyes across multiple directions, with the exception of the superotemporal, upper, and central regions.

In terms of CT, CSC-affected eyes exhibited significantly higher values compared to healthy eyes across the majority of subfields (*P* < 0.05). Specifically, the upper, temporal, central, inferotemporal, and lower regions in CSC-affected eyes notably exceeded the CT values of healthy eyes. Conversely, when compared to fellow eyes, no significant differences in CT were observed across all subfields in CSC-affected eyes.

Notably, RT in CSC-affected eyes exhibited significantly higher values than healthy eyes across most subfields, with the central area being the sole exception, where RT was reduced. Particularly, the superotemporal and superonasal regions notably exceeded the RT values of healthy eyes. Conversely, when compared to fellow eyes, no significant differences in RT were observed across all subfields in CSC-affected eyes.

### Comparison of choroidal parameter and retinal thickness in nine subfields in the acute CSC-affected eyes, the chronic CSC-affected eyes, and healthy eyes

**Fig. 3:**
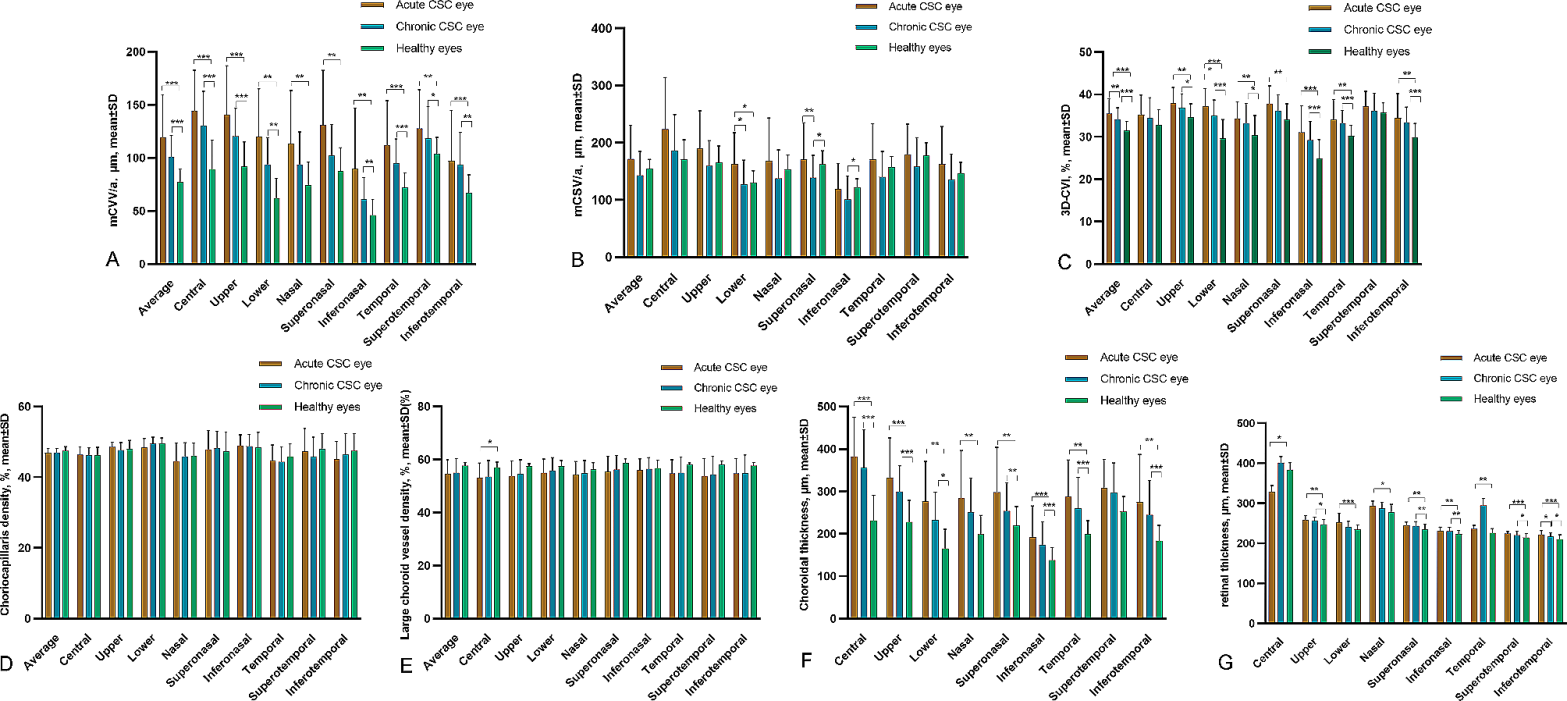
Choroidal characteristics and retinal thickness were assessed in nine subfields among eyes with acute CSC, chronic CSC, and healthy eyes (**A**) Choroidal vessel volume per unit area (mCVV/a); (**B**) Choroidal stroma volume per unit area (mCSV/a); (**C**) Three-dimensional choroidal vascularity index (3D-CVI); (**D**) Choriocapillaris density; (**E**) Large choroid vessel density; (**F**) Choroidal thickness; (**G**) Retinal thickness.∗*P* < 0.05; ∗∗*P* < 0.01; ∗∗∗*P* < 0.001

**Fig. 4:**
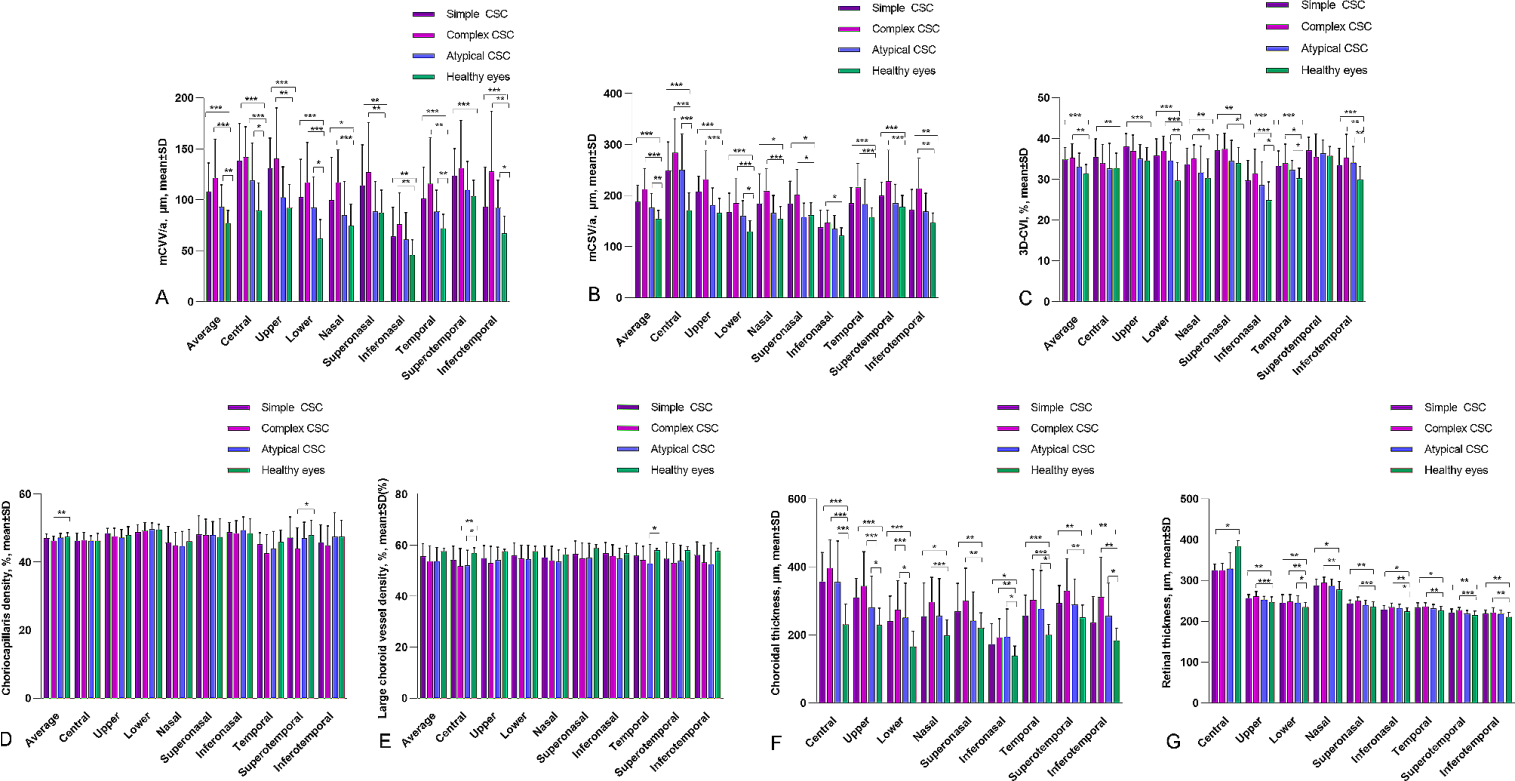
Choroidal characteristics and retinal thickness in nine subfields of healthy eyes, eyes with simple CSC, complex CSC, and atypical CSC (**A**) Choroidal vessel volume per unit area (mCVV/a); (**B**) Choroidal stroma volume per unit area (mCSV/a); (**C**) Three-dimensional choroidal vascularity index (3D-CVI); (**D**) Choriocapillaris density; (**E**) Large choroid vessel density; (**F**) Choroidal thickness; (**G**) Retinal thickness. ∗*P* < 0.05; ∗∗*P* < 0.01; ∗∗∗*P* < 0.001

The findings depicted in Fig. 2 conclusively suggest that fellow eyes are not adequate as healthy controls. As a result, in Figs. 3 and 4, exclusively healthy eyes were utilized as controls. In Fig. 3, the most widely adopted clinical classification was employed to compare choroidal parameters and RT across nine subfields within acute CSC, chronic CSC, and healthy eyes (for detailed information, refer to Supplementary Table 2).

The mean mCVV/a values for acute CSC, chronic CSC, and healthy eyes were 119.46 ± 40.03 μm, 100.94 ± 20.28 μm, and 77.03 ± 12.6 μm, respectively. In acute CSC-affected eyes, mCVV/a significantly exceeded values in healthy eyes across all subfields (*P* < 0.01). Chronic CSC exhibited elevated mCVV/a values compared to healthy eyes in all regions except for the superotemporal area. Additionally, mCVV/a in the lower region was higher in acute CSC than in chronic CSC.

The mean mCSV/a values for acute CSC, chronic CSC, and healthy eyes were recorded as 171.92 ± 58.51 μm, 142.51 ± 42.33 μm, and 153.83 ± 16.92 μm, respectively. Acute CSC displayed increased mCSV/a values compared to healthy eyes in the lower region. Conversely, chronic CSC exhibited decreased mCSV/a in the superonasal and inferonasal regions when compared with healthy eyes.

The mean 3D-CVI values for acute CSC, chronic CSC, and healthy eyes were determined as 35.42 ± 3.6 μm, 34.09 ± 2.76 μm, and 31.45 ± 2.15 μm, respectively. Both acute and chronic CSC-affected eyes exhibited elevated 3D-CVI values in various regions when compared to healthy eyes.

The average values for vascular density in the large choroidal vessel layer in acute CSC, chronic CSC, and healthy eyes were 54.58 ± 5.3 μm, 55.06 ± 5.27 μm, and 57.62 ± 1.24 μm, respectively. Notably, the central region of acute CSC had decreased vascular density when compared to healthy eyes.

CT in acute CSC-affected eyes consistently exceeded that of healthy eyes across all subfields. In chronic CSC, CT was notably higher than in healthy eyes, particularly in the superonasal region.

RT in acute CSC exhibited variations compared to healthy eyes, with increased thickness observed in most regions, except for the central area. Chronic CSC displayed distinct variations in RT in specific regions compared to healthy eyes. However, no significant difference was noted when comparing RT across all subfields between acute and chronic CSC.

### Comparison of choroidal parameter and retinal thickness in nine subfields in simple CSC, complex CSC-affected eyes, atypical eyes, and healthy eyes

Figure 4 depicts a novel multimodal imaging-based classification system for CSC, as proposed by the CSC International Group in 2020, addressing previous limitations. This figure presents a detailed breakdown of nine subfields pertaining to choroidal parameters, including CT, choriocapillaris density, vascular density of the large choroidal vessel layer, 3D-CVI, mCVV/a, mCSV/a, and RT, for simple CSC, complex CSC, atypical CSC, and healthy eyes (for detailed data, please refer to Supplementary Table 3).

The mean mCVV/a values observed for simple CSC, complex CSC, atypical CSC, and healthy eyes were 107.6 ± 28.72 μm, 121.64 ± 37.85 μm, 93.1 ± 21.68 μm, and 77.03 ± 12.6 μm, respectively. In all nine subfields, the mCVV/a for simple CSC significantly exceeded that of the healthy eyes (*P* < 0.01). For the complex CSC group, significant variations were noted in the temporal, inferotemporal, upper, central, lower, superonasal, nasal, inferonasal, and average regions (*P* < 0.01). At the same time, the atypical CSC group presented higher mCVV/a values than healthy eyes in specific regions, including the temporal, central, inferotemporal, upper, and average regions.

The mean mCSV/a values for simple CSC, complex CSC, atypical CSC, and healthy eyes were 187.4 ± 33.15 μm, 212.64 ± 40.24 μm, 176 ± 28.14 μm, and 153.83 ± 16.92 μm, respectively. In all nine subfields, both the simple and complex CSC groups exhibited mCSV/a values significantly higher than those of the healthy eyes (*P* < 0.01). For the atypical CSC group, elevated mCSV/a values were observed in the central, upper, and average regions when compared to healthy eyes.

The mean 3D-CVI values observed in the simple CSC, complex CSC, atypical CSC, and healthy eyes were 34.83 ± 2.96 μm, 35.21 ± 3.49 μm, 33.1 ± 3.28 μm, and 31.45 ± 2.15 μm, respectively. In the simple CSC group, 3D-CVI values were significantly elevated compared to those in healthy eyes across the superotemporal, temporal, inferotemporal, upper, central, lower, superonasal, nasal, inferonasal, and average regions (*P* < 0.01). In the complex CSC group, increased 3D-CVI values were noted in the inferotemporal, upper, lower, superonasal, nasal, and inferonasal regions when compared to healthy eyes. Furthermore, the atypical CSC group demonstrated higher 3D-CVI values in the inferotemporal and lower regions than the healthy eyes, and the differences were statistically significant (*P* < 0.05).

The mean vascular density values of the large choroidal vessel layer for the simple CSC, complex CSC, atypical CSC, and healthy eyes were 55.63 ± 4.93 μm, 53.79 ± 5.89 μm, 53.7 ± 5.46 μm, and 57.62 ± 1.24 μm, respectively. In the complex CSC group, the vascular density in the central region was notably lower at 51.79 ± 6.9 μm, compared to 56.97 ± 2.08 μm in healthy eyes (*P* = 0.009). Similarly, for the atypical CSC group, the vascular density was diminished in both the temporal (52.8 ± 7.48 μm vs. 58.03 ± 0.68 μm, *P* = 0.031) and central (52.1 ± 5.95 μm vs. 56.97 ± 2.08 μm, *P* = 0.021) regions in comparison to healthy eyes.

The CT across all 9 subfields for both simple CSC and complex CSC-affected eyes was significantly greater than that observed in healthy eyes (*P* < 0.01). For the atypical CSC group, the CT was notably elevated in the temporal, inferotemporal, upper, central, and superonasal regions when compared to healthy eyes, with these differences being statistically significant (*P* < 0.01).

For both the simple CSC and complex CSC groups, RT was found to be higher than that in healthy eyes across several regions, namely superotemporal, temporal, inferotemporal, upper, lower, superonasal, nasal, and inferonasal. However, in the central region, RT was less than in healthy eyes, with these variations proving statistically significant (*P* < 0.01). For the atypical CSC category, RT was notably increased in the lower (245.4 ± 16.71 μm compared to 234.55 ± 11.32 μm, *P* = 0.027) and inferonasal (194.4 ± 81.89 μm compared to 138.17 ± 29.07 μm, *P* = 0.020) regions compared to healthy eyes.

### ROC curves of choroidal parameter and retinal thickness in nine subfields in acute CSC and chronic CSC

**Fig. 5:**
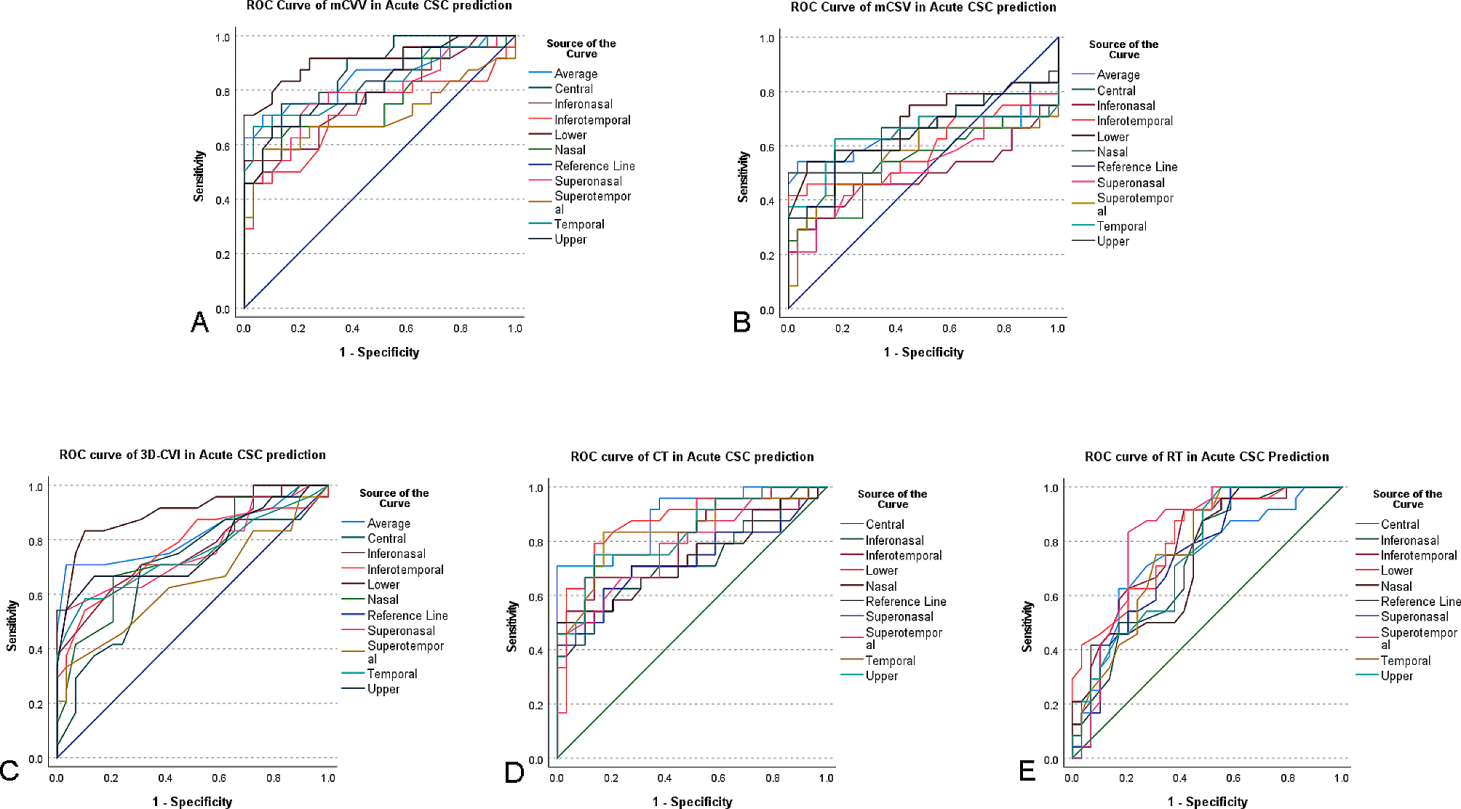
Receiver operating characteristic (ROC) curve analysis of acute CSC

**Fig. 6:**
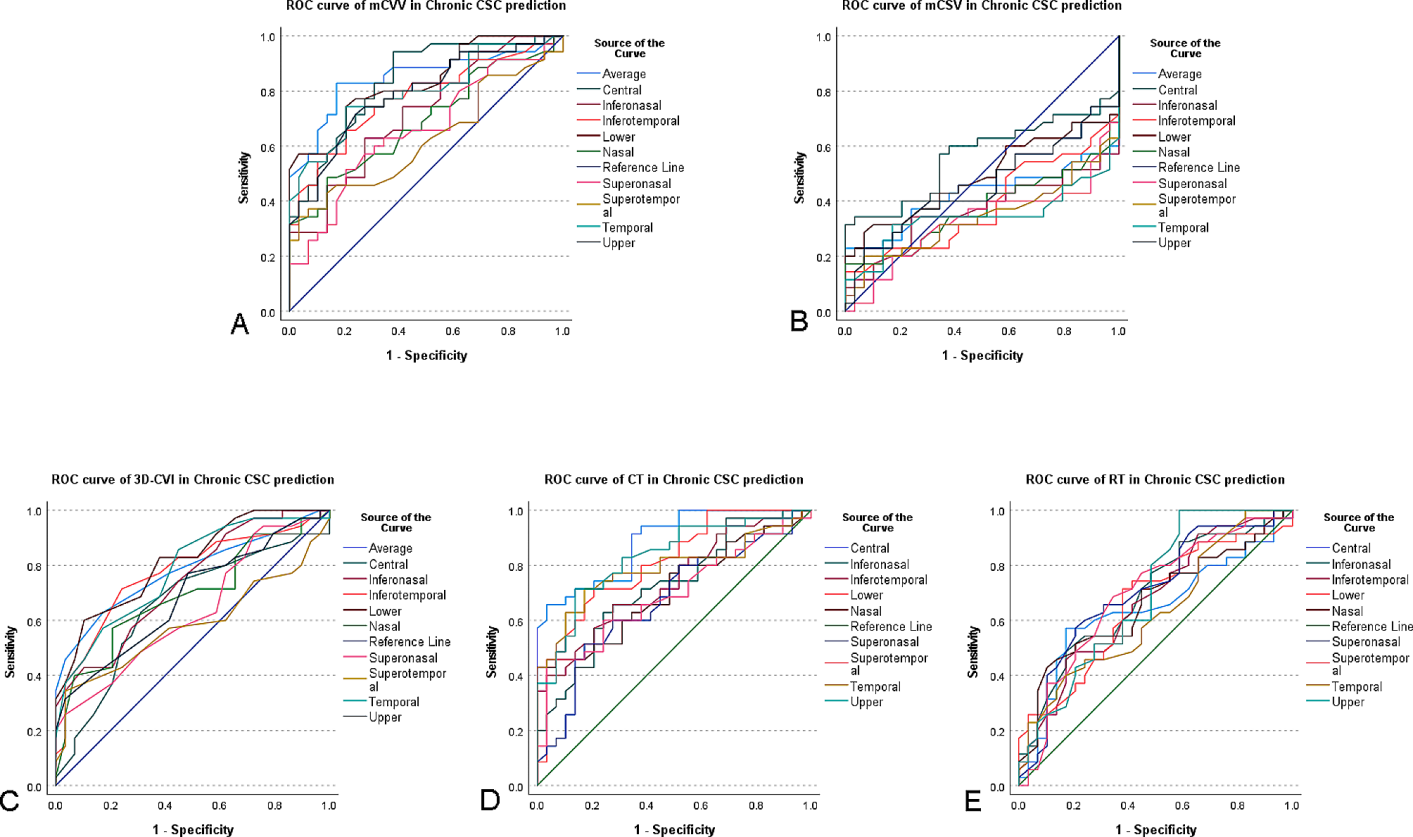
Receiver operating characteristic (ROC) curve analysis of chronic CSC

We employed ROC curves to assess the diagnostic efficacy of choroidal and retinal parameters derived from SS-OCTA in distinguishing between acute and chronic CSC. Our previous findings indicated significant differences in mCVV/a, mCSV/a, 3D-CVI, CT, and RT among acute CSC, chronic CSC, and healthy eyes. To further enhance our diagnostic insights for acute and chronic CSC, we examined ROC curves for these parameters across their respective nine subfields, as detailed in Figs. 5 and 6 (refer to Supplementary Table 4 for specific values).

For acute CSC, the ROC curve analysis revealed the following AUC values for mCVV/a across the nine subfields, listed in descending order: lower (0.913), central (0.878), average (0.848), temporal (0.825), upper (0.819), inferonasal (0.789), superonasal (0.786), nasal (0.755), inferotemporal (0.713), and supratemporal (0.705).

In the acute CSC analysis, the AUC values for mCSV/a in all 9 subfields and the median were found to be below 0.7 based on the ROC curve analysis.

In the acute CSC analysis, the AUC values for the 3D-CVI in 9 subfields, ranked from highest to lowest, were as follows: lower (0.904), average (0.819), lower temporal (0.766), upper (0.764), inferonasal (0.763), superonasal (0.757), temporal (0.746), nasal (0.739), central (0.687), and superior temporal (0.639).

In terms of RT in acute CSC across the nine subfields, the AUC values, in descending order, were: central (0.889), lower (0.879), temporal (0.843), upper (0.832), inferotemporal (0.793), superotemporal (0.774), nasal (0.737), superonasal (0.731), and inferonasal (0.724).

Regarding CT in acute CSC, the AUC values in descending order across the nine subfields were: superotemporal (0.822), lower (0.807), inferotemporal (0.787), lateral temporal (0.766), upper (0.746), superonasal (0.740), central (0.737), nasal (0.736), and inferonasal (0.722).

For chronic CSC, the AUC values for mCVV/a across the nine subfields and average, arranged from highest to lowest, were: central (0.836), lower (0.832), upper (0.790), average (0.848), inferotemporal (0.774), lateral temporal (0.798), superotemporal (0.624), inferonasal (0.717), superonasal (0.659), and nasal (0.687).

For mCSV/a in chronic CSC, all AUC values were below 0.5.

In the case of 3D-CVI in chronic CSC, the descending AUC values across the nine subfields were: lower (0.813), temporal (0.779), average (0.779), inferotemporal (0.771), inferonasal (0.739), nasal (0.690), upper (0.684), and central (0.653).

Regarding CT in chronic CSC, the AUC values in descending order were: central (0.885), upper (0.828), lower (0.806), temporal (0.787), inferotemporal (0.738), inferonasal (0.722), nasal (0.711), superotemporal (0.693), and superonasal (0.671).

For the chronic CSC analysis of CT across the nine subfields, the AUC for superonasal reached 0.704, while all other directions yielded values below 0.7.

## Discussion

This cross-sectional study indicate that both CSC-affected eyes and their fellow eyes exhibit significant elevations in parameters such as mCVV/a, mCSV/a, 3D-CVI, CT, and RT when compared to healthy eyes. These variations, especially prominent in specific retinal subfields, offer potential insights into the mechanisms behind choroidal hyperpermeability in CSC. mCVV/a and 3D-CVI serve as metrics delineating the vascular and blood flow constituents within the choroidal vascular layer. Disparities observed in the retinal subfields between CSC-affected eyes and those of healthy counterparts in mCVV/a and mCVV/a reveal a notable augmentation in choroidal vascular and stromal elements in CSC, notably accentuated in the temporal superior, nasal superior, central, and upper regions. This asymmetrical spatial distribution may explain the heightened choroidal permeability and functional deficits characteristic of CSC.

These findings align with previous research conducted by Zeng et al. [15]., which also noted an elevated 3D-CVI in CSC-affected eyes across various subfields using UWF SS-OCTA. Nevertheless, some discrepancies exist between the results, which may be attributed to differences in inclusion criteria and disease durations. Interestingly, our results, in line with the findings of Izumi et al., indicate that CSC can manifest bilaterally with monocular symptoms [15]. While previous research indicated a decline in choriocapillaris density in CSC-affected eyes, our results differ, possibly due to our extended imaging range and the specific regions analyzed [15]. Further investigation is warranted to mitigate potential discrepancies stemming from variations in measurement methodologies. Furthermore, our findings, like those of Izumi, highlight significant choroidal thickening in CSC-affected eyes compared to healthy eyes [15]. Our analysis also revealed reduced RT in most subfields of CSC-affected eyes compared to healthy eyes, possibly due to prolonged CSC or recurrent retinal fluid episodes.

Previous investigations have predominantly used central retinal thickness as a benchmark for assessing treatment efficacy in patients with CSC, often neglecting its use as a quantitative measure for examining CSC in fellow and healthy eyes [6, 16–18]. In our study, we used RT as a numerical parameter in a cross-sectional analysis. This approach not only facilitated an assessment of macular region thickness but also provided insights into broader retinal thickness across adjacent areas. Interestingly, we noted a noteworthy elevation in RT in the periphery of CSC-affected eyes compared to healthy counterparts. However, RT in the macular region of CSC-affected eyes was observed to be lower than that in healthy eyes, notwithstanding higher RT values in various orientations compared to fellow eyes. Although these differences did not attain statistical significance, likely due to sample size limitations, it is imperative to expand the sample size to delve deeper into the underlying reasons.

Notably, thickness disparities were observed between fellow eyes and healthy eyes, indicating potential systemic risk factors for CSC. This underscores the need for long-term monitoring of fellow eyes and implies that future research should prioritize healthy eyes over fellow eyes for control comparisons to better standardize the disease model.

While previous research has identified the dilation of the large choroidal vessel layer as a key pathogenic mechanism in CSC, limited investigations have compared choroidal parameter changes between acute and chronic CSC [15]. Our findings demonstrate that parameters such as mCVV/a, 3D-CVI, CT, and RT are notably higher in acute CSC compared to healthy eyes, consistent with earlier studies [15]. Despite chronic CSC exhibiting higher mCSV/a values in most regions compared to healthy eyes, a reduction was observed in the central region, indicating a decrease in the stromal content in chronic CSC. This aligns with the findings of Minsub et al. [15] They proposed that this could be attributed to chronic low-grade inflammation stemming from persistent venous congestion, leading to subsequent tissue atrophy. In our cross-sectional study, RT served as the primary metric of analysis. We observed that, in CSC-affected eyes, only the central region of RT did not achieve statistical significance when compared to fellow eyes. This discrepancy may be attributed to the sample size constraints inherent in our study, emphasizing the necessity for larger sample sizes in future research to foster a more thorough comprehension of the pathogenic mechanisms.

Choroidal thickness within the central macular region demonstrates its highest values in acute CSC, chronic CSC, and healthy eyes, potentially attributed to the elevated metabolic demands of the macula, which relies solely on choroidal blood supply [19]. Conversely, peripheral regions (temporal > nasal > superior > inferior) exhibit a significant decrease in choroidal thickness when compared to the central macular area, with the temporal inferior region displaying the thinnest thickness. In chronic CSC, a statistically significant reduction in choroidal thickness is noted in all directions compared to acute CSC. While the precise etiology of the disease remains elusive, Spaide et al. hypothesized that fluid leakage could result from restricted outflow, indicating a potential association between congested and anastomosed vortex veins and CSC [20]. Our study revealed a significant increase in CVI across superior, inferior, nasal, temporal, and central locations in acute CSC compared to chronic CSC and healthy eyes (*P* < 0.05), validating the uneven distribution of choroidal blood flow. Consequently, we speculate that the initial phase of CSC may involve factors leading to excessive choroidal perfusion, followed by an anatomically unbalanced drainage system, resulting in an uneven distribution of venous blood flow. Congestion of vortex veins may prompt choroidal thickening, with venous-venous anastomoses potentially forming over time between different vortex veins, thereby reducing choroidal congestion and subsequently diminishing choroidal thickness. Further investigations are warranted to validate this hypothesis.

When taken collectively, significant differences exist between acute and chronic CSC, particularly in the lower regions of mCVV/a, mCSV/a, and 3D-CVI. The underlying causes of these differences require further investigation in comprehensive future studies.

To date, no studies have assessed choroidal parameters in UWF SS-OCTA using the new multimodal imaging classification system, making our study a pioneer in employing this approach. We observed that parameters such as mCSV/a, CT, and RT in both simple and complex CSC were significantly elevated compared to healthy eyes. However, the increase in simple CSC was less pronounced than that in complex CSC. Conversely, atypical CSC demonstrated predominantly significant differences, indicating that the inclusion of other retinal diseases within the atypical CSC category might contribute to concerns regarding sample size adequacy. This observation implies that the magnitude of elevation could serve as a guiding factor in clinical diagnosis, potentially facilitating the differentiation between simple, complex, and atypical CSC manifestations.

While future comprehensive studies with larger samples are needed to validate these findings, our research, despite its limited sample size, provides valuable insights into choroidal and retinal parameter changes using the new multimodal imaging classification for CSC.

There is no universally accepted standard that designates CSC as a chronic disease, even though a disease duration of 3 to 6 months is often cited in clinical practice [21]. Such a distinction is crucial as the pathophysiology of acute CSC may differ from that of chronic CSC, necessitating different treatment approaches. To address this, we employed ROC curves to examine the diagnostic utility of various choroidal parameters and RT in SS-OCTA for both acute and chronic CSC. Our findings revealed that mCVV/a, 3D-CVI, RT, and CT offered superior diagnostic efficacy for acute CSC, while for chronic CSC, mCVV/a and CT revealed the highest efficacy, followed by 3D-CVI and RT. Historically, CT has been a key diagnostic parameter for CSC, but it is noteworthy that some CSC cases do not present significant CT changes [21]. As CSC research has evolved, CVI has emerged as a reliable indicator, with growing evidence pointing to choroidal vascular changes as a contributor to the pathology of CSC [21]. This underscores the clinical relevance of indicators such as 3D-CVI, CVV, mCVV/a, and RT.

Our study has certain limitations. Primarily, the cross-sectional design presents inherent constraints. Moreover, it is important to note that our study utilized a limited sample size, particularly in the context of the new multimodal imaging classification, wherein atypical CSC cases comprised only 10 eyes. This restriction in sample size has implications for the robustness of our findings. This necessitates an expansion in the sample size for a more comprehensive examination of differences between various CSC classifications. Additionally, certain parameters, such as choriocapillaris density and vascular density of the large choroidal vessel layer, did not exhibit statistical correlation across subgroups. This may stem from the limited sample size rather than indicating a genuine absence of correlation. A deeper understanding of choroidal changes in CSC would benefit from future prospectives, longitudinal studies, which could track choroidal changes throughout different disease phases. Nonetheless, despite these challenges, our research offers fresh perspectives on the pathogenesis across different CSC classifications and the diagnostic approaches for both acute and chronic CSC.

## Conclusion

SS-OCTA emerges as a sophisticated imaging modality with significant usage in the identification and diagnostic assessment of retinal disorders. Our study has delineated discernible variations in SS-OCTA metrics among healthy eyes, CSC-affected eyes, and subcategories of acute and chronic CSC, using a novel multimodal classification system. By concurrently analyzing both retinal and choroidal parameters, our findings underscore the potential enhancement of clinical diagnostic strategies for CSC. Furthermore, the exploration of parameter differentials across distinct retinal subfields offers valuable insights into the anatomical nuances of varied CSC classifications. This meticulous examination of subfield characteristics may furnish a deeper understanding of the heterogeneous nature of CSC presentations, thereby fostering further inquiry into the diverse phenotypic manifestations of the condition.

### Electronic supplementary material

Below is the link to the electronic supplementary material.

Supplementary Material 1

## Data Availability

The datasets generated and/or analysed during the current study are not publicly available due [REASON WHY DATA ARE NOT PUBLIC] but are available from the corresponding author on reasonable request.

## Abbreviations

UWF SS-OCTA: Ultra‑wide field swept‑source optical coherence tomography angiography
CSC: Central serous chorioretinopathy
CT: Choroidal thickness
3D-CVI: Three-dimensional choroidal vascularity index
mCVV/a: Choroidal vessel volume per unit area
mCSV/a: Choroidal stroma volume per unit area
RT: Retinal thickness
RPE: Retinal pigment epithelium
SRF: Subretinal fluid
OCTA: Optical coherence tomography angiography
OCT: Optical coherence tomography
PED: Pigment epithelial detachment
ICGA: Indocyanine green angiography
FFA: Fundus fluorescein angiography
ILM: Inner limiting membrane
OPL: Outer plexiform layer
BCVA: Best-corrected visual acuity
