# Supplementary material for: Quantitative assessment of choroidal parameters and retinal thickness in central serous chorioretinopathy using ultra-widefield swept-source optical coherence tomography: a cross-sectional study
Source: BMC Ophthalmol. 2024 Apr 17;24:176. doi: 10.1186/s12886-024-03405-w (PMC11025137; doi:10.1186/s12886-024-03405-w)
Supplement: Supplementary file 1 — Supplementary Material 1 [file 12886_2024_3405_MOESM1_ESM.docx]

**Supplementary Table 1.** Choroidal parameters and Retina thickness in eyes with CSC, fellow and healthy eyes

|  | CSC eyes | Fellow eyes | Control eyes | P1 | P2 | P3 |
| --- | --- | --- | --- | --- | --- | --- |
| **mCVV/a，μm, mean±SD** | |  |  |  |  |  |
| Average | 107.68±32.12 | 97.06±20.88 | 77.03±12.6 | 0.7 | ＜0.001 | ＜0.001 |
| Central | 138.35±36.79 | 123.44±36.64 | 89.45±27.09 | 0.58 | ＜0.001 | 0.001 |
| Upper | 130.44±38.42 | 116.16±26.06 | 92.03±23.07 | 0.423 | ＜0.001 | 0.005 |
| Lower | 104.15±37.18 | 91.53±27.95 | 62.03±18.62 | 0.68 | ＜0.001 | ＜0.001 |
| Nasal | 100.85±43.2 | 90.41±26.73 | 74.31±21.68 | 0.041 | 0.001 | 0.041 |
| Superonasal | 112.32±46.64 | 105.47±28.64 | 87.34±22.17 | 1 | 0.003 | 0.014 |
| Inferonasal | 64.32±28.12 | 57.94±18.88 | 45.86±14.94 | 1 | ＜0.001 | 0.025 |
| Temporal | 99.79±34.42 | 91.16±27.12 | 71.9±13.91 | 0.784 | ＜0.001 | 0.002 |
| Superotemporal | 124.15±32.58 | 110±24.79 | 103.52±15.74 | 0.309 | 0.007 | 0.674 |
| Inferotemporal | 93.91±39.07 | 87.38±30.69 | 66.9±17.03 | 1 | ＜0.001 | 0.003 |
| **mCSV/a，μm, mean±SD** | |  |  |  |  |  |
| Average | 187.5±36.04 | 176.31±26.82 | 153.83±16.92 | 0.302 | ＜0.001 | 0.003 |
| Central | 251.82±57.95 | 228.63±65.74 | 170.83±34.49 | 0.141 | ＜0.001 | 0.001 |
| Upper | 205.88±41.13 | 193.03±32.88 | 165.45±29.1 | 0.162 | ＜0.001 | 0.02 |
| Lower | 169.38±40.05 | 155.56±29.09 | 129.45±21.5 | 0.369 | ＜0.001 | 0.003 |
| Nasal | 185.44±54.62 | 170.31±34.5 | 153.62±24.89 | 0.905 | 0.008 | 0.229 |
| Superonasal | 179.65±46.29 | 176.44±31.04 | 161.69±24.27 | 1 | 0.04 | 0.211 |
| Inferonasal | 136.38±25.87 | 129.28±17.67 | 121.76±15.19 | 0.794 | 0.017 | 0.407 |
| Temporal | 187.09±37.81 | 178.38±30.59 | 157.52±18.33 | 0.575 | ＜0.001 | 0.019 |
| Superotemporal | 199.97±36.34 | 191.84±44.04 | 177.76±22.32 | 0.173 | 0.004 | 0.652 |
| Inferotemporal | 172.26±43.49 | 163.56±30.04 | 146.52±19.47 | 0.612 | 0.001 | 0.095 |
| **3D-CVI, %, mean±SD** | |  |  |  |  |  |
| Average | 34.65±3.4 | 34±2.55 | 31.45±2.15 | 0.313 | ＜0.001 | ＜0.001 |
| Central | 35.18±4.37 | 34.75±3.53 | 32.79±3.62 | 1 | 0.025 | 0.102 |
| Upper | 37.76±3.53 | 36.84±2.34 | 34.62±3.19 | 0.087 | 0.003 | 0.087 |
| Lower | 35.91±4.18 | 34.94±3.75 | 29.66±4.39 | 1 | ＜0.001 | ＜0.001 |
| Nasal | 33.47±5.25 | 33.19±3.96 | 30.34±4.68 | 1 | 0.004 | 0.041 |
| Superonasal | 37±4.61 | 36.38±3.74 | 34.03±3.78 | 1 | 0.012 | 0.041 |
| Inferonasal | 29.53±5.65 | 29.16±4.52 | 24.9±4.46 | 0.509 | ＜0.001 | ＜0.001 |
| Temporal | 33.35±3.95 | 32.59±3.43 | 30.24±2.49 | 0.186 | ＜0.001 | 0.008 |
| Superotemporal | 36.97±3.16 | 35.53±3.87 | 35.72±2.3 | 0.156 | 0.156 | 0.156 |
| Inferotemporal | 33.32±4.42 | 32.72±5.66 | 29.9±3.23 | 0.283 | ＜0.001 | 0.02 |
| **Choriocapillaris density, %, mean±SD** | |  |  |  |  |  |
| Average | 46.91±1.14 | 47.69±1.53 | 47.45±1.24 | 0.018 | 0.251 | 1 |
| Central | 46±2.17 | 46.25±2.95 | 46.31±2.16 | 0.821 | 0.916 | 0.92 |
| Upper | 48±1.86 | 48.28±3.12 | 47.97±2.44 | 0.399 | 0.399 | 0.399 |
| Lower | 48.79±2.19 | 49.44±1.64 | 49.59±1.55 | 0.871 | 0.871 | 0.871 |
| Nasal | 43.74±4.82 | 46±3.89 | 46.14±3.55 | 0.851 | 0.851 | 0.851 |
| Superonasal | 46.06±5.15 | 48.34±5.65 | 47.41±5.37 | 0.559 | 0.559 | 0.559 |
| Inferonasal | 48.85±2.88 | 47.81±4.07 | 48.52±4.22 | 0.561 | 0.561 | 0.561 |
| Temporal | 45.68±4.09 | 46.25±3.44 | 45.93±3.53 | 0.091 | 0.091 | 0.091 |
| Superotemporal | 49.35±4.4 | 48.59±4.26 | 48.03±4.29 | 0.272 | 0.272 | 0.272 |
| Inferotemporal | 45.74±5.79 | 48.25±4.15 | 47.48±4.84 | 0.182 | 0.182 | 0.182 |
| **Large choroid vessel density, %, mean±SD(%)** | | |  |  |  |  |
| Average | 55.71±4.92 | 56.47±4.19 | 57.62±1.24 | 0.534 | 0.534 | 0.534 |
| Central | 54±5.47 | 55.13±4.82 | 56.97±2.08 | 0.673 | 0.031 | 0.664 |
| Upper | 55.12±4.95 | 56.03±4.51 | 57.59±0.82 | 0.228 | 0.228 | 0.228 |
| Lower | 56.32±4.77 | 56.63±4.28 | 57.55±2.08 | 0.958 | 0.958 | 0.958 |
| Nasal | 55.09±4.66 | 56.19±4.07 | 56.38±2.41 | 0.276 | 0.276 | 0.276 |
| Superonasal | 56.91±5.36 | 57.53±4.08 | 58.79±1.47 | 0.54 | 0.54 | 0.54 |
| Inferonasal | 56.91±3.8 | 57.5±3.66 | 56.79±2.97 | 0.29 | 0.29 | 0.29 |
| Temporal | 55.68±5.53 | 56.72±4.41 | 58.03±0.68 | 0.265 | 0.265 | 0.265 |
| Superotemporal | 55.15±5.8 | 56.22±5.43 | 58±1.46 | 0.267 | 0.267 | 0.267 |
| Inferotemporal | 55.79±6.41 | 56.53±4.44 | 57.69±1.14 | 0.692 | 0.692 | 0.692 |
| **Choroidal thickness, μm, mean±SD** | |  |  |  |  |  |
| Central | 365.53±95.95 | 322.56±96.82 | 230.9±59.95 | 0.186 | ＜0.001 | 0.001 |
| Upper | 314.65±81.72 | 279.75±57.76 | 228.1±50.88 | 0.173 | ＜0.001 | 0.008 |
| Lower | 252.29±86.78 | 217.63±56.61 | 165.52±45.06 | 0.445 | ＜0.001 | 0.003 |
| Nasal | 266.97±103.32 | 231.22±59.78 | 198.59±45.05 | 0.494 | 0.002 | 0.215 |
| Superonasal | 269.88±94.85 | 252.34±58.59 | 219.55±45.01 | 1 | 0.007 | 0.084 |
| Inferonasal | 179.5±62.91 | 160.94±42.1 | 138.17±29.07 | 0.778 | 0.002 | 0.132 |
| Temporal | 267.82±84.18 | 240.13±56.6 | 199.97±31.05 | 0.346 | ＜0.001 | 0.01 |
| Superotemporal | 301.62±68.77 | 272.38±58.82 | 251.86±36.06 | 0.126 | 0.002 | 0.578 |
| Inferotemporal | 243.68±91.06 | 221.38±58.56 | 183.83±35.81 | 0.597 | ＜0.001 | 0.031 |
| **retinal thickness, μm, mean±SD** | |  |  |  |  |  |
| Central | 322.38±23.77 | 321.53±13.52 | 383.48±372.87 | 0.474 | 0.015 | 0.607 |
| Upper | 256.06±9.14 | 254.84±8.3 | 246.72±12.87 | 0.968 | 0.002 | 0.089 |
| Lower | 245.79±19.97 | 240.75±9.62 | 234.55±11.32 | 0.346 | 0.001 | 0.239 |
| Nasal | 290.15±13.22 | 284.13±16.14 | 278.03±19.28 | 0.475 | 0.006 | 0.38 |
| Superonasal | 243±8.94 | 240.88±9.78 | 235.17±12.46 | 0.566 | 0.001 | 0.131 |
| Inferonasal | 230.71±7.62 | 228.34±8.79 | 223.34±9.33 | 0.251 | 0.001 | 0.038 |
| Temporal | 233.15±10.11 | 232.41±13.27 | 226.31±10.75 | 0.639 | 0.011 | 0.399 |
| Superotemporal | 221.24±8.61 | 221.81±7.3 | 214.52±10.14 | 1 | ＜0.001 | 0.005 |
| Inferotemporal | 217.88±7.99 | 217±7.78 | 210.62±11 | 1 | 0.001 | 0.06 |

**Supplementary Table 2.** Choroidal parameters and Retina thickness in eyes with acute CSC, chronic CSC and healthy eyes

|  | Acute CSC eye | Chronic CSC eye | Control eyes | *P1* | *P2* | *P3* |
| --- | --- | --- | --- | --- | --- | --- |
| **mCVV/a，μm, mean±SD** | |  |  |  |  |  |
| Average | 119.46±40.03 | 100.94±20.28 | 77.03±12.6 | ＜0.001 | ＜0.001 | 0.126 |
| Central | 144.29±38.21 | 130.31±32.49 | 89.45±27.09 | ＜0.001 | ＜0.001 | 0.738 |
| Upper | 140.17±46.61 | 120.31±26.4 | 92.03±23.07 | ＜0.001 | ＜0.001 | 0.186 |
| Lower | 119.71±45.43 | 93.8±25.21 | 62.03±18.62 | ＜0.001 | ＜0.001 | 0.047 |
| Nasal | 113.17±50.24 | 93.83±30.6 | 74.31±21.68 | 0.004 | 0.012 | 0.279 |
| Superonasal | 130.79±51.64 | 102.43±29.02 | 87.34±22.17 | 0.004 | 63 | 0.103 |
| Inferonasal | 89.79±57.07 | 60.89±20.4 | 45.86±14.94 | 0.004 | 0.017 | 1 |
| Temporal | 111.96±41.99 | 94.86±22.93 | 71.9±13.91 | ＜0.001 | ＜0.001 | 1 |
| Superotemporal | 127.67±36.55 | 118.29±29.97 | 103.52±15.74 | 0.005 | 0.043 | 0.445 |
| Inferotemporal | 97±47.8 | 93.31±30.94 | 66.9±17.03 | ＜0.001 | 0.001 | 1 |
| **mCSV/a，μm, mean±SD** | |  |  |  |  |  |
| Average | 171.92±58.51 | 142.51±42.33 | 153.83±16.92 | 0.089 | 0.089 | 0.089 |
| Central | 223.79±89.95 | 186.03±63.16 | 170.83±34.49 | 0.101 | 0.101 | 0.101 |
| Upper | 190.58±65.27 | 159.03±44.31 | 165.45±29.1 | 0.243 | 0.865 | 0.13 |
| Lower | 162.83±54.59 | 127.6±42.06 | 129.45±21.5 | 0.025 | 0.994 | 0.032 |
| Nasal | 168.08±75.07 | 137.49±50.04 | 153.62±24.89 | 0.747 | 0.268 | 0.24 |
| Superonasal | 170.67±64.19 | 138.66±39.61 | 161.69±24.27 | 0.462 | 0.04 | 0.007 |
| Inferonasal | 118.75±44.84 | 100.43±41.22 | 121.76±15.19 | 0.985 | 0.02 | 0.31 |
| Temporal | 170.79±62.51 | 140.17±44.52 | 157.52±18.33 | 0.682 | 0.117 | 0.129 |
| Superotemporal | 179.92±52.43 | 157.94±50.87 | 177.76±22.32 | 0.06 | 0.06 | 0.06 |
| Inferotemporal | 162.08±66.19 | 135.4±44.87 | 146.52±19.47 | 0.611 | 0.468 | 0.251 |
| **3D-CVI, %, mean±SD** | |  |  |  |  |  |
| Average | 35.42±3.6 | 34.09±2.76 | 31.45±2.15 | ＜0.001 | ＜0.001 | 0.081 |
| Central | 35.13±4.73 | 34.37±4.86 | 32.79±3.62 | 0.056 | 0.11 | 1 |
| Upper | 37.92±3.73 | 36.8±3.33 | 34.62±3.19 | 0.002 | 0.04 | 0.713 |
| Lower | 37.29±4.09 | 34.97±3.7 | 29.66±4.39 | ＜0.001 | ＜0.001 | 0.033 |
| Nasal | 34.33±3.94 | 33.23±4.68 | 30.34±4.68 | 0.007 | 0.03 | 1 |
| Superonasal | 37.83±4.17 | 36.09±3.86 | 34.03±3.78 | 0.004 | 0.197 | 0.307 |
| Inferonasal | 31±6.35 | 29.2±4.39 | 24.9±4.46 | ＜0.001 | ＜0.001 | 0.179 |
| Temporal | 34.08±4.74 | 33.2±2.97 | 30.24±2.49 | 0.003 | ＜0.001 | 0.802 |
| Superotemporal | 37.29±3.42 | 36.09±4.2 | 35.72±2.3 | 0.2 | 0.2 | 0.2 |
| Inferotemporal | 34.38±5.84 | 33.34±3.67 | 29.9±3.23 | 0.006 | ＜0.001 | 0.827 |
| **Choriocapillaris density, %, mean±SD** | |  |  |  |  |  |
| Average | 46.88±1.3 | 46.97±1.22 | 47.45±1.24 | 0.156 | 0.156 | 0.156 |
| Central | 46.42±2.22 | 46.31±2 | 46.31±2.16 | 0.856 | 0.994 | 0.855 |
| Upper | 48.58±1.35 | 47.69±2.14 | 47.97±2.44 | 0.378 | 0.378 | 0.378 |
| Lower | 48.42±2.57 | 49.46±1.84 | 49.59±1.55 | 0.2 | 0.2 | 0.2 |
| Nasal | 44.54±5.12 | 45.94±3.84 | 46.14±3.55 | 0.63 | 0.63 | 0.63 |
| Superonasal | 47.92±5.36 | 48.26±4.72 | 47.41±5.37 | 0.832 | 0.832 | 0.832 |
| Inferonasal | 48.96±3.04 | 48.77±3.33 | 48.52±4.22 | 0.992 | 0.992 | 0.992 |
| Temporal | 44.63±4.48 | 44.31±4.28 | 45.93±3.53 | 0.293 | 0.293 | 0.293 |
| Superotemporal | 47.33±6.49 | 45.77±5.61 | 48.03±4.29 | 0.226 | 0.226 | 0.226 |
| Inferotemporal | 45.04±5.15 | 46.49±5.88 | 47.48±4.84 | 0.198 | 0.198 | 0.198 |
| **Large choroid vessel density, %, mean±SD(%)** | | |  |  |  |  |
| Average | 54.58±5.3 | 55.06±5.27 | 57.62±1.24 | 0.818 | 0.818 | 0.818 |
| Central | 52.96±5.7 | 53.57±6.01 | 56.97±2.08 | 0.022 | 0.136 | 1 |
| Upper | 53.79±5.7 | 54.66±5.24 | 57.59±0.82 | 0.296 | 0.296 | 0.296 |
| Lower | 55±5.17 | 55.86±4.86 | 57.55±2.08 | 0.64 | 0.64 | 0.64 |
| Nasal | 54.21±5.05 | 54.89±4.66 | 56.38±2.41 | 0.67 | 0.67 | 0.67 |
| Superonasal | 55.42±5.79 | 56.23±5.25 | 58.79±1.47 | 0.456 | 0.456 | 0.456 |
| Inferonasal | 55.92±4.33 | 56.49±4.19 | 56.79±2.97 | 0.853 | 0.853 | 0.853 |
| Temporal | 54.79±5.12 | 55±5.88 | 58.03±0.68 | 0.57 | 0.57 | 0.57 |
| Superotemporal | 53.83±6.53 | 54.29±6.92 | 58±1.46 | 0.353 | 0.353 | 0.353 |
| Inferotemporal | 54.75±5.62 | 54.91±6.77 | 57.69±1.14 | 0.855 | 0.855 | 0.855 |
| **Choroidal thickness, μm, mean±SD** | |  |  |  |  |  |
| Central | 382.46±92.13 | 355.66±90.09 | 230.9±59.95 | ＜0.001 | ＜0.001 | 0.932 |
| Upper | 331.33±95.06 | 299.34±60.8 | 228.1±50.88 | ＜0.001 | ＜0.001 | 0.388 |
| Lower | 276.46±94.12 | 231.97±66.71 | 165.52±45.06 | ＜0.001 | ＜0.001 | 0.411 |
| Nasal | 284.33±112.31 | 251±80.49 | 198.59±45.05 | 0.004 | 0.02 | 1 |
| Superonasal | 298.25±105.67 | 253.57±66.65 | 219.55±45.01 | 0.006 | 0.087 | 0.73 |
| Inferonasal | 191.75±73.77 | 173.71±54.61 | 138.17±29.07 | 0.007 | 0.013 | 1 |
| Temporal | 287.13±86.75 | 259.51±73.11 | 199.97±31.05 | ＜0.001 | ＜0.001 | 1 |
| Superotemporal | 307.5±67.61 | 297.43±69.27 | 251.86±36.06 | 0.003 | 0.017 | 1 |
| Inferotemporal | 274.75±112.73 | 244.29±81.24 | 183.83±35.81 | ＜0.001 | 0.005 | 1 |
| **retinal thickness, μm, mean±SD** | |  |  |  |  |  |
| Central | 328.38±16.03 | 401.2±458.55 | 383.48±372.87 | 0.017 | 0.11 | 1 |
| Upper | 258.67±10.1 | 256.03±9.25 | 246.72±12.87 | 0.004 | 0.03 | 1 |
| Lower | 252.5±22.64 | 241.6±13.5 | 234.55±11.32 | ＜0.001 | 0.091 | 0.139 |
| Nasal | 293.04±12.78 | 286.97±15.83 | 278.03±19.28 | 0.01 | 0.069 | 1 |
| Superonasal | 245.04±8.26 | 243.54±10.7 | 235.17±12.46 | 0.001 | 0.003 | 0.6 |
| Inferonasal | 231.54±8.98 | 230.09±10.03 | 223.34±9.33 | 0.002 | 0.006 | 0.566 |
| Temporal | 236.5±8.59 | 294.63±370.35 | 226.31±10.75 | 0.004 | 0.201 | 0.303 |
| Superotemporal | 224.54±5.45 | 220.69±9.35 | 214.52±10.14 | ＜0.001 | 0.044 | 0.144 |
| Inferotemporal | 222.13±9.57 | 217.03±9.59 | 210.62±11 | ＜0.001 | 0.013 | 0.06 |

**Supplementary Table 3.** Choroidal parameters and Retina thickness in eyes with simple CSC, complex CSC ,atypical CSC and healthy eyes

|  | Simple | Complex | Atypical | Control eyes | *P1* | *P2* | *P3* |
| --- | --- | --- | --- | --- | --- | --- | --- |
| **mCVV/a，μm, mean±SD** | |  |  |  |  |  |  |
| Average | 107.6±28.72 | 121.64±37.85 | 93.1±21.68 | 77.03±12.6 | ＜0.001 | ＜0.001* | 0.007* |
| Central | 138.37±36.53 | 142±29.77 | 119.3±36.33 | 89.45±27.09 | ＜0.001 | ＜0.001 | 0.02 |
| Upper | 131.03±29.6 | 140.5±49.69 | 102.2±30.26 | 92.03±23.07 | ＜0.001 | 0.003* | 0.275 |
| Lower | 102.57±37.12 | 117.29±38.96 | 92.4±30.13 | 62.03±18.62 | ＜0.001 | ＜0.001 | 0.012* |
| Nasal | 99.57±42.09 | 117.14±32.01 | 84.5±33.41 | 74.31±21.68 | 0.01 | ＜0.001 | 0.386* |
| Superonasal | 114.09±39.81 | 127.21±48.47 | 88.6±29.38 | 87.34±22.17 | 0.003 | 0.004 | 0.888* |
| Inferonasal | 64.11±28.45 | 75.86±30.83 | 60.9±26.54 | 45.86±14.94 | 0.005 | 0.003* | 0.117* |
| Temporal | 100.86±30.98 | 115.93±45.15 | 88.4±21.07 | 71.9±13.91 | ＜0.001 | 0.003* | 0.008 |
| Superotemporal | 123.89±26.33 | 131±46.84 | 109.8±27.84 | 103.52±15.74 | ＜0.001 | 0.177 | 0.383* |
| Inferotemporal | 93.03±39.13 | 127.71±59.14 | 92.1±27.14 | 66.9±17.03 | ＜0.001 | 0.002 | 0.018* |
| **mCSV/a，μm, mean±SD** | |  |  |  |  |  |  |
| Average | 187.4±33.15 | 212.64±40.24 | 176±28.14 | 153.83±16.92 | ＜0.001 | ＜0.001 | 0.005 |
| Central | 248.6±56.6 | 284.21±66.11 | 249.7±71.48 | 170.83±34.49 | ＜0.001 | ＜0.001 | ＜0.001 |
| Upper | 207.66±30.76 | 231.86±55.94 | 181±33.75 | 165.45±29.1 | ＜0.001 | ＜0.001* | 0.170* |
| Lower | 167.71±36.97 | 185.29±47.82 | 159.5±30.22 | 129.45±21.5 | ＜0.001 | ＜0.001 | 0.013 |
| Nasal | 184.57±58.18 | 208.36±44.24 | 165.5±34.68 | 153.62±24.89 | 0.03 | ＜0.001 | 0.248 |
| Superonasal | 184.57±43.67 | 201.57±50 | 157.8±27.46 | 161.69±24.27 | 0.011 | 0.012 | 0.675 |
| Inferonasal | 137.77±33.42 | 146.5±24.88 | 134.8±26.27 | 121.76±15.19 | 0.05 | 0.03 | 0.165 |
| Temporal | 184.91±30.68 | 215.57±47.12 | 182.8±49.6 | 157.52±18.33 | ＜0.001 | ＜0.001 | 0.147* |
| Superotemporal | 199.37±27.23 | 228.29±60.82 | 185±37.2 | 177.76±22.32 | 0.001 | 0.001 | 0.573 |
| Inferotemporal | 171.49±40.18 | 213.5±60.46 | 168.7±35.91 | 146.52±19.47 | 0.005 | 0.001* | 0.11 |
| **3D-CVI, %, mean±SD** |  |  |  |  |  |  |  |
| Average | 34.83±2.96 | 35.21±3.49 | 33.1±3.28 | 31.45±2.15 | ＜0.001* | 0.002* | 0.077* |
| Central | 35.54±4.35 | 33.93±4.57 | 32.7±6.11 | 32.79±3.62 | 0.009 | 0.381 | 0.965 |
| Upper | 38±3.18 | 36.93±3.93 | 35.1±3.38 | 34.62±3.19 | ＜0.001 | 0.07 | 0.73 |
| Lower | 35.86±4.04 | 37±3.57 | 34.6±4.35 | 29.66±4.39 | ＜0.001 | ＜0.001 | 0.004* |
| Nasal | 33.69±3.96 | 35.07±3.38 | 31.7±6.41 | 30.34±4.68 | 0.003 | 0.003 | 0.38 |
| Superonasal | 37.2±3.68 | 37.43±3.88 | 34.5±5.04 | 34.03±3.78 | 0.002 | 0.017 | 0.97 |
| Inferonasal | 29.74±4.91 | 31.43±5.91 | 28.5±5.8 | 24.9±4.46 | ＜0.001* | ＜0.001* | 0.049* |
| Temporal | 33.37±3.58 | 33.93±4.71 | 32.3±2.31 | 30.24±2.49 | ＜0.001 | 0.014 | 0.028 |
| Superotemporal | 37.09±3.22 | 35.5±5.63 | 36.3±3.3 | 35.72±2.3 | 0.061 | 0.888 | 0.546 |
| Inferotemporal | 33.46±4.09 | 35.29±5.74 | 34.1±3.9 | 29.9±3.23 | ＜0.001 | 0.004 | 0.002* |
| **Choriocapillaris density, %, mean±SD** | |  |  |  |  |  |  |
| Average | 47.11±1.16 | 46.29±1.33 | 47.2±1.23 | 47.45±1.24 | 0.21 | 0.007 | 0.52 |
| Central | 46.31±2.21 | 46.5±2.24 | 46.3±1.42 | 46.31±2.16 | 0.994 | 0.791 | 0.989* |
| Upper | 48.46±1.46 | 47.64±2.31 | 47.2±2.39 | 47.97±2.44 | 0.553 | 0.732 | 0.37 |
| Lower | 48.77±2.2 | 49.21±2.46 | 49.7±1.89 | 49.59±1.55 | 0.227 | 0.801 | 0.77 |
| Nasal | 45.74±4.75 | 44.93±3.81 | 44.7±4.3 | 46.14±3.55 | 0.776 | 0.313 | 0.302* |
| Superonasal | 48.2±5.43 | 48±4.61 | 48±3.94 | 47.41±5.37 | 0.409 | 0.886 | 0.96 |
| Inferonasal | 48.83±2.82 | 48.5±3.67 | 49.4±3.92 | 48.52±4.22 | 0.946 | 1 | 0.57 |
| Temporal | 45.29±3.39 | 42.64±5.51 | 44±5.03 | 45.93±3.53 | 0.279 | 0.067 | 0.5 |
| Superotemporal | 47.2±6.13 | 44±6.11 | 47±4.71 | 48.03±4.29 | 0.957 | 0.039* | 0.525* |
| Inferotemporal | 45.83±5.14 | 44.93±5.72 | 47.5±7.09 | 47.48±4.84 | 0.255 | 0.134 | 0.42 |
| **Large choroid vessel density, %, mean±SD(%)** | |  |  |  |  |  |  |
| Average | 55.63±4.93 | 53.79±5.89 | 53.7±5.46 | 57.62±1.24 | 0.65 | 0.277 | 0.1 |
| Central | 54.29±5.32 | 51.79±6.9 | 52.1±5.95 | 56.97±2.08 | 0.089 | 0.009 | 0.02 |
| Upper | 54.91±4.97 | 52.93±6.64 | 54.1±5.13 | 57.59±0.82 | 0.32 | 0.335 | 0.21 |
| Lower | 56.06±4.81 | 54.86±5.17 | 54.5±5.44 | 57.55±2.08 | 0.709 | 0.497 | 0.27 |
| Nasal | 55.14±4.59 | 54±5.56 | 53.6±4.55 | 56.38±2.41 | 0.995 | 0.487 | 0.11 |
| Superonasal | 56.57±5.16 | 54.79±6.14 | 55.1±5.61 | 58.79±1.47 | 0.656 | 0.183 | 0.18 |
| Inferonasal | 56.86±4.21 | 55.79±4.39 | 54.8±3.99 | 56.79±2.97 | 0.4 | 0.638 | 0.17 |
| Temporal | 55.83±4.86 | 54.14±5.49 | 52.8±7.48 | 58.03±0.68 | 0.717 | 0.159 | 0.03 |
| Superotemporal | 54.6±6.63 | 53±7.66 | 53.9±6.05 | 58±1.46 | 0.283 | 0.475 | 0.16 |
| Inferotemporal | 56.17±5.11 | 53.21±6.83 | 52.5±8.41 | 57.69±1.14 | 0.15 | 0.175 | 0.19 |
| **Choroidal thickness, μm, mean±SD** | |  |  |  |  |  |  |
| Central | 357.43±84.97 | 396.71±83.16 | 356.3±119.86 | 230.9±59.95 | ＜0.001 | ＜0.001 | 0 |
| Upper | 309.26±57.35 | 343.14±101.72 | 280.1±92.95 | 228.1±50.88 | ＜0.001 | 0.001 | 0.032* |
| Lower | 240.77±73.33 | 273.21±85.9 | 250.2±102.19 | 165.52±45.06 | ＜0.001 | ＜0.001 | 0.029 |
| Nasal | 254.69±98.32 | 296.07±72.99 | 255±110.59 | 198.59±45.05 | 0.019 | ＜0.001 | 0.148 |
| Superonasal | 269.26±82.37 | 299.5±96.91 | 241.6±83.92 | 219.55±45.01 | 0.005 | 0.006 | 0.298 |
| Inferonasal | 172.57±60.83 | 192.71±54.63 | 194.4±81.89 | 138.17±29.07 | 0.015 | 0.003 | 0.03 |
| Temporal | 256.49±60.34 | 302.14±90.34 | 276.7±112.82 | 199.97±31.05 | ＜0.001 | ＜0.001 | 0.02 |
| Superotemporal | 293.83±51.4 | 329.93±93.84 | 288.7±74.83 | 251.86±36.06 | 0.001 | 0.009 | 0.21 |
| Inferotemporal | 235.11±77.9 | 311.64±117.28 | 255.2±97.87 | 183.83±35.81 | 0.001 | 0.001 | 0.03 |
| **retinal thickness, μm, mean±SD** | |  |  |  |  |  |  |
| Central | 324.63±15.27 | 324.5±17.55 | 328.8±38.97 | 383.48±372.87 | 0.019 | 0.06 | 0.23 |
| Upper | 256.6±8.59 | 261.5±11.52 | 252.7±8.47 | 246.72±12.87 | 0.004 | ＜0.001* | 0.181* |
| Lower | 245.4±19.62 | 248.07±17.51 | 245.4±16.71 | 234.55±11.32 | 0.005 | 0.004* | 0.027* |
| Nasal | 288.31±14.74 | 294.43±13.74 | 286.4±16.59 | 278.03±19.28 | 0.012 | 0.006 | 0.21 |
| Superonasal | 243.34±8.39 | 249.43±9.74 | 239.6±11.75 | 235.17±12.46 | 0.004 | ＜0.001 | 0.332 |
| Inferonasal | 228.91±9.06 | 234.29±10.19 | 231.8±9.78 | 223.34±9.33 | 0.019 | 0.001 | 0.020 |
| Temporal | 233.89±11.43 | 236.64±8.71 | 230.9±10.22 | 226.31±10.75 | 0.017 | 0.003 | 0.246* |
| Superotemporal | 221.49±8.22 | 226.79±6.78 | 218.6±7.69 | 214.52±10.14 | 0.001 | ＜0.001 | 0.254 |
| Inferotemporal | 218.49±9.37 | 221.36±11.57 | 218.1±9.33 | 210.62±11 | 0.003 | 0.005 | 0.062 |

*P<0.05

**Supplementary Table 4.** Acute CSC, chronic CSC choroidal characteristics and retinal thickness AUC based on ROC curve analysis

|  |  | **Acute CSC** |  | **Chronic CSC** |  |
| --- | --- | --- | --- | --- | --- |
|  |  | AUC | *P* | AUC | *P* |
| **mCVV/a** | Average | 0.85 | ＜0.001 | 0.85 | ＜0.001 |
|  | Central | 0.88 | ＜0.001 | 0.84 | ＜0.001 |
|  | Upper | 0.83 | ＜0.001 | 0.79 | ＜0.001 |
|  | Lower | 0.91 | ＜0.001 | 0.83 | ＜0.001 |
|  | Nasal | 0.76 | 0.002 | 0.69 | 0.01 |
|  | Superonasal | 0.79 | ＜0.001 | 0.66 | 0.03 |
|  | Inferonasal | 0.91 | ＜0.001 | 0.72 | 0.003 |
|  | Temporal | 0.83 | ＜0.001 | 0.8 | ＜0.001 |
|  | Superotemporal | 0.71 | 0.011 | 0.62 | 0.091 |
|  | Inferotemporal | 0.71 | 0.008 | 0.77 | ＜0.001 |
| **mCVV/a** | Average | 0.64 | 0.09 | 0.42 | 0.284 |
|  | Central | 0.67 | 0.041 | 0.56 | 0.384 |
|  | Upper | 0.64 | 0.083 | 0.45 | 0.526 |
|  | Lower | 0.69 | 0.17 | 0.49 | 0.882 |
|  | Nasal | 0.54 | 0.611 | 0.37 | 0.081 |
|  | Superonasal | 0.53 | 0.721 | 0.33 | 0.02 |
|  | Inferonasal | 0.49 | 0.922 | 0.37 | 0.067 |
|  | Temporal | 0.64 | 0.091 | 0.35 | 0.034 |
|  | Superotemporal | 0.56 | 0.464 | 0.34 | 0.033 |
|  | Inferotemporal | 0.59 | 0.249 | 0.38 | 0.109 |
| **3D-CVI** | Average | 0.819 | ＜0.001 | 0.779 | ＜0.001 |
|  | Central | 0.687 | 0.02 | 0.653 | 0.037 |
|  | Upper | 0.764 | 0.001 | 0.684 | 0.012 |
|  | Lower | 0.904 | ＜0.001 | 0.813 | ＜0.001 |
|  | Nasal | 0.739 | 0.003 | 0.69 | 0.009 |
|  | Superonasal | 0.757 | 0.001 | 0.635 | 0.065 |
|  | Inferonasal | 0.763 | 0.001 | 0.739 | 0.001 |
|  | Temporal | 0.746 | 0.002 | 0.779 | ＜0.001 |
|  | Superotemporal | 0.64 | 0.083 | 0.59 | 0.246 |
|  | Inferotemporal | 0.766 | 0.001 | 0.771 | ＜0.001 |
| **Choroidal thickness（CT）** | Central | 0.889 | ＜0.001 | 0.885 | ＜0.001 |
|  | Upper | 0.832 | ＜0.001 | 0.828 | ＜0.001 |
|  | Lower | 0.879 | ＜0.001 | 0.806 | ＜0.001 |
|  | Nasal | 0.737 | 0.03 | 0.711 | 0.004 |
|  | Superonasal | 0.731 | 0.004 | 0.671 | 0.019 |
|  | Inferonasal | 0.724 | 0.005 | 0.722 | 0.002 |
|  | Temporal | 0.843 | ＜0.001 | 0.787 | ＜0.001 |
|  | Superotemporal | 0.774 | 0.001 | 0.693 | 0.008 |
|  | Inferotemporal | 0.793 | ＜0.001 | 0.738 | 0.001 |
| **retinal thickness（RT）** | Central | 0.737 | 0.003 | 0.641 | 0.053 |
|  | Upper | 0.746 | 0.002 | 0.695 | 0.008 |
|  | Lower | 0.807 | ＜0.001 | 0.661 | 0.028 |
|  | Nasal | 0.736 | 0.003 | 0.665 | 0.024 |
|  | Superonasal | 0.74 | 0.003 | 0.704 | 0.005 |
|  | Inferonasal | 0.722 | ＜0.001 | 0.692 | 0.009 |
|  | Temporal | 0.766 | 0.001 | 0.629 | 0.077 |
|  | Superotemporal | 0.822 | ＜0.001 | 0.693 | 0.008 |
|  | Inferotemporal | 0.787 | ＜0.001 | 0.671 | 0.019 |
